# ITK independent development of Th17 responses during hypersensitivity pneumonitis driven lung inflammation

**DOI:** 10.1038/s42003-022-03109-1

**Published:** 2022-02-24

**Authors:** Jessica Elmore, Chavez Carter, Amie Redko, Nicholas Koylass, Amelia Bennett, Max Mead, Marinel Ocasio-Rivera, Weishan Huang, Ankur Singh, Avery August

**Affiliations:** 1 Department of Microbiology & Immunology, Cornell Center for Immunology, Cornell Institute for Host Microbe-Interactions and Disease, Cornell University, Ithaca, NY USA; 2 Department of Pathobiological Sciences, School of Veterinary Medicine, Louisiana State University, Baton Rouge, LA USA; 3 Nancy E & Peter C Meinig School of Biomedical Engineering and Department of Mechanical & Aerospace Engineering, Cornell University, Ithaca, NY USA; 4 Cornell Center for Health Equity, Cornell University, Ithaca, NY USA; 5 Present Address: Scientis Pharma Inc., NY, NY USA; 6 Present Address: LEO Pharma Inc., Tampa, FL USA; 7 Present Address: Johns Hopkins University School of Medicine, Baltimore, MD USA; 8 Present Address: University of California, Davis, CA USA; 9 Present Address: University of Puerto Rico, Rio Piedras, PR USA; 10 Present Address: Coulter Department of Biomedical Engineering, Georgia Tech, Atlanta, GA USA

**Keywords:** Immunology, T cells

## Abstract

T helper 17 (Th17) cells develop in response to T cell receptor signals (TCR) in the presence of specific environments, and produce the inflammatory cytokine IL17A. These cells have been implicated in a number of inflammatory diseases and represent a potential target for ameliorating such diseases. The kinase ITK, a critical regulator of TCR signals, has been shown to be required for the development of Th17 cells. However, we show here that lung inflammation induced by *Saccharopolyspora rectivirgula* (SR) induced Hypersensitivity pneumonitis (SR-HP) results in a neutrophil independent, and ITK independent Th17 responses, although ITK signals are required for γδ T cell production of IL17A. Transcriptomic analysis of resultant ITK independent Th17 cells suggest that the SR-HP-induced extrinsic inflammatory signals may override intrinsic T cell signals downstream of ITK to rescue Th17 responses in the absence of ITK. These findings suggest that the ability to pharmaceutically target ITK to suppress Th17 responses may be dependent on the type of inflammation.

## Abstract (web summary)

The kinase Itk is normally required for differentiation of IL-17-producing CD4 T-helper (Th)17 cells however, Elmore, Carter et al. now report that Itk can be bypassed during Th17 differentiation in Saccharopolyspora rectivirgula induced hypersensitivity pneumonitis. This study suggests that some inflammatory signals can override normal T cell activation and differentiation pathways.

## Introduction

The CD4^+^ effector Th17 cells play important roles in the immune response to infection at mucosal surfaces. They have also been implicated in the development of a number of diseases including asthma, hypersensitivity pneumonitis (HP), and autoimmune diseases such as rheumatoid arthritis^1–7^. Th17 cells express the transcription factor RAR–related Orphan Receptor gamma T (RORγt) and secrete the inflammatory cytokine IL17 among other cytokines (IL17A, IL17F, IL21, and IL22^8^). Th17 cells differentiate following activation by the TCR in the presence of cytokines such as IL6 and TGFβ^8^, or IL21 and TGFβ^9^. Although the signaling pathways downstream of the TCR that drives the development of Th17 cells remain unclear, we and others have shown that the TCR-activated tyrosine kinase ITK is critical. ITK plays an important role as an amplifier of the TCR signals, and in humans, and/or mouse models, has been shown to regulate the development of both CD4^+^ and CD8^+^ T-cells in the thymus, and influences the development of specific populations of γδ T cells, *i*NKT cells, and intestinal Innate Lymphoid Cell 2 populations^10–16^. In the periphery, the signals regulated by ITK are able to tune the development of CD8^+^ T cell memory cells^17^, as well as the differentiation of CD4^+^ effector T cells^18–23^. A specific role for ITK in the development of Th17 cells has been demonstrated in both mouse models^24^, and in human T cells^11,25^. This conclusion is supported by the finding that humans with mutations in ITK resulting in *Itk* deficiency have reduced proportions of Th17 cells^11^. A better understanding of the development and function of Th17 cells, including the role of the pharmaceutically tractable ITK in the process, would further the development of therapeutic options for inflammatory diseases driven by IL17.

HP, Farmer’s Lung, a type of HP, is caused by repeated inhalation of foreign emissions, dust, or residues in moldy hay, specifically the thermophilic bacteria *Saccharopolyspora rectivirgula* (SR)^26–28^. The development of SR-induced HP includes a prominent role for Th17 cytokine IL17A^27,29,30^. Here, we have used IL17A-GFP reporter mice to clearly define cellular sources of IL17A during the development of SR-HP and used these mice to determine the role of ITK in the development of Th17-driven SR-HP. We show that γδ and later CD4^+^ αβ T cells are the predominant sources of IL17A during SR-induced development of HP. Furthermore, contrary to previous reports, we find that neutrophils are not major producers of this cytokine in vivo in this disease, nor are they required for the development of Th17 response in the lung. In addition, we found that surprisingly, ITK is not required for the development and cytokine production of IL17A by Th17 cells in the SR-induced Th17-driven lung inflammation in HP. Transcriptomic and chromatin accessibility analysis revealed little difference between WT and *Itk*^*−/−*^ Th17 cells, although inflammation-related genes were upregulated in the absence of ITK. These data suggest that ITK differentially regulates Th17 cytokine production dependent on the type of lung inflammation, and has implications for understanding T helper differentiation programs of T cell-driven inflammatory diseases.

## Results

### γδ and αβ T cells, not neutrophils, are the major producers of IL17A in the lung, and Tec family kinase ITK is required for γδ T cell, but not αβ T cell production of IL17A during the development of SR-HP

**Fig. 1:**
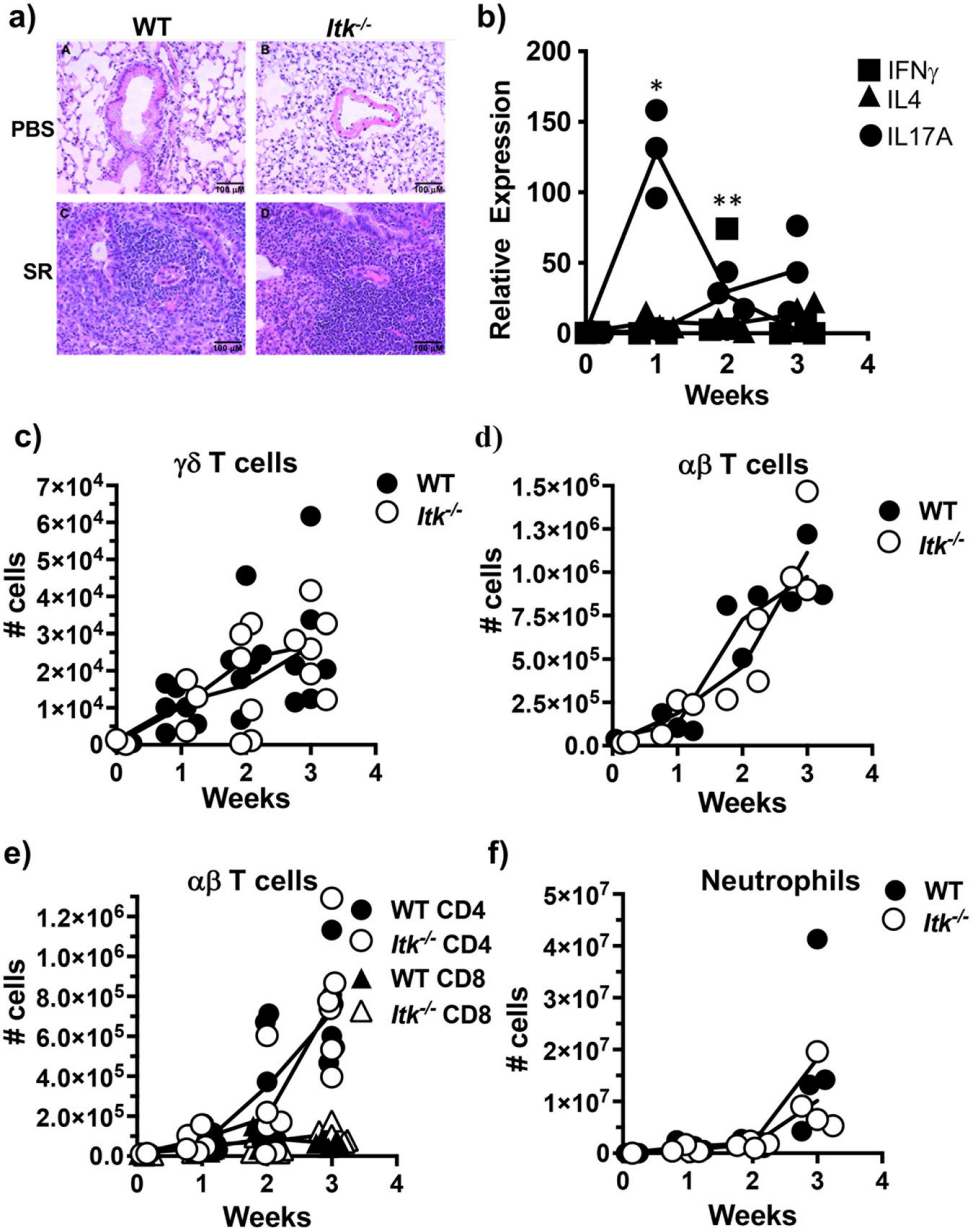
ITK is not required for the development of SR-induced hypersensitivity pneumonitis. **a** WT or *Itk*^*−/−*^ mice were exposed to PBS or SR intranasally 3×/week over 3 weeks and lung sections were analyzed by H&E staining. **b** Lungs from SR-exposed WT mice were analyzed for the indicated mRNA by qRT-PCR (filled squares: IFNγ; filled triangles: IL4 and filled circles: IL17A, *n* = 3, *p = 0.0025 vs. time 0, **p = 0.007 vs. time 0 for IL17A). **c** WT or *Itk*^*−/−*^ mice were exposed to SR as in (**a**) and lungs analyzed for total cell numbers of γδ T cells (filled circles: WT γδ T cells; open circles *Itk*^*−/−*^ γδ T cells, *n* = 6 for WT group; 3–6 for *Itk*^*−/−*^ group), **d** αβ T cells (filled circles: WT αβ T cells; open circles *Itk*^*−/−*^ αβ T cells, (*n* = 3/group), **e** CD4^+^ and CD8^+^ T cells (filled circles: WT CD4^+^ T cells; open circles *Itk*^*−/−*^ CD4^+^ T cells; filled triangles: WT CD8^+^ T cells; open triangles *Itk*^*−/−*^ CD8^+^ T cells, (*n* = 3–6/group), or **f** neutrophils (filled circles: WT; open circles *Itk*^*−/−*^, *n* = 3–4/group).

Previous groups have shown that SR induces a predominant IL17A response, which drives lung inflammation^27,29,30^, however, the source of IL17A in SR-HP remains unexamined. Our experiments confirmed this conclusion that SR exposure induces lung inflammation, accompanied by high levels of IL17A in the lungs of WT mice, with little change in IFNγ and IL4 (Fig. 1b). Analysis of lungs of WT mice during the development of SR-HP over a 3-week time course revealed accumulation of γδ, CD4^+^ and CD8^+^ αβ T cells, and neutrophils (Fig. 1a–f).

ITK has been shown to be required for the development of Th17 cells in humans and mice, including in a murine model of allergic asthma^22,24^. To determine the role of ITK in SR-HP, we analyzed the development of lung inflammation following exposure to SR in WT and *Itk*^*−/−*^ mice. We found that unexpectedly, *Itk*^*−/−*^ mice exhibited similar inflammation and immune cell infiltration as WT mice following exposure to SR (Fig. 1a–f, Supplementary Fig. 1a). Furthermore, like WT mice, lungs from *Itk*^*−/−*^ mice had substantial levels of mRNA for IL17A, but not IFNγ, as a result of SR exposure, and no difference in expression of IL17F, IL4, and IL13 between WT and *Itk*^*−/−*^ mice (Supplementary Fig. 1b, c). Analysis of lungs during the development of SR-HP over a 3-week time course revealed that the absence of ITK did not affect the accumulation of γδ T cells, CD4^+^, CD8^+^, αβ T cells or neutrophils (Fig. 1c–f). Given the fact that SR-induced HP has been shown to be dependent on IL17A^30^, these data suggest that contrary to expectations, ITK may not be required to generate a Th17 mediated inflammatory response in the lung in response to SR exposure.

**Fig. 2:**
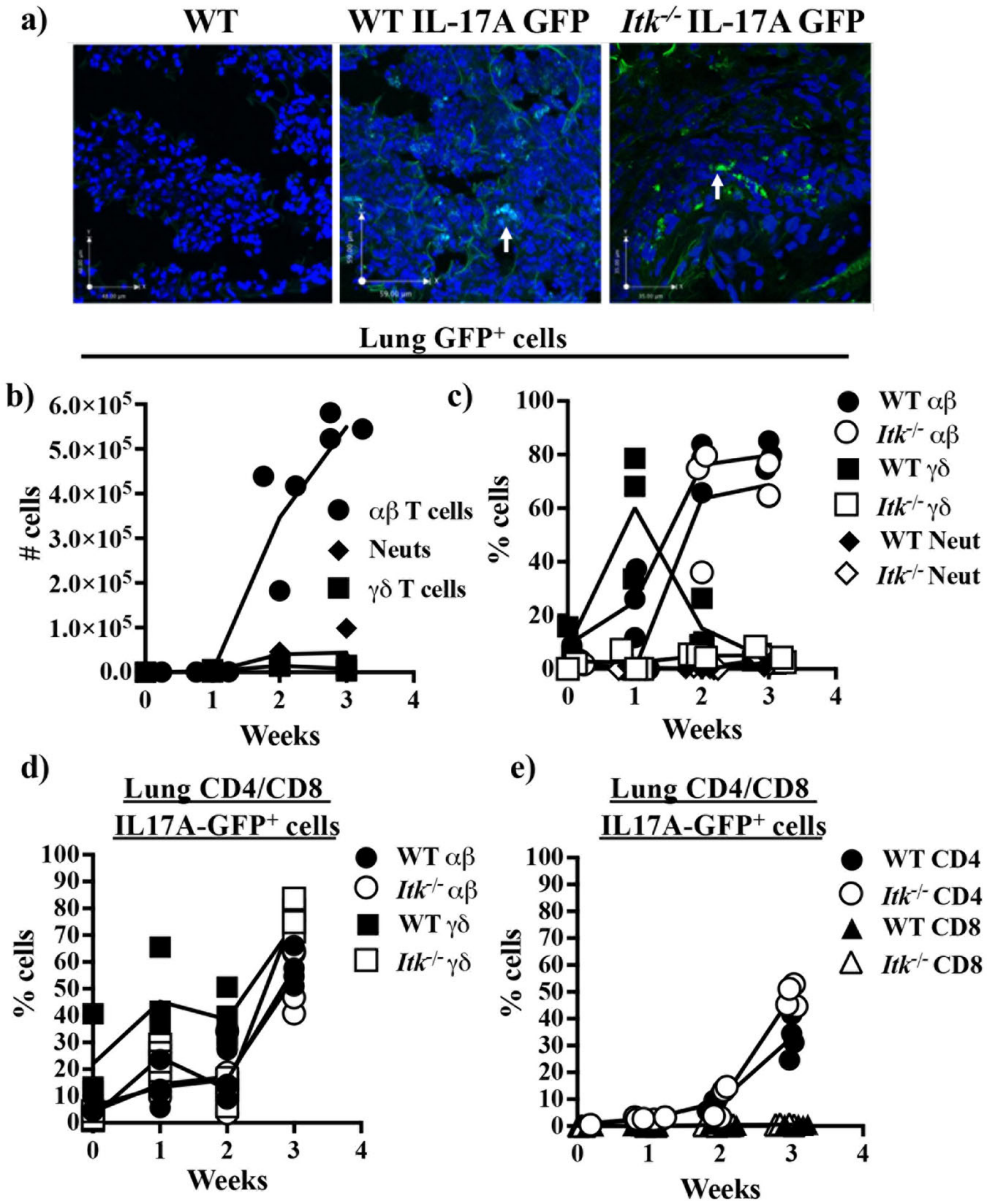
αβ T cells are the major producers of IL17A during the development of SR-induced hypersensitivity pneumonitis and ITK is not required for their ability to produce IL17A. **a** WT non-IL17A-GFP reporter mice, or WT or *Itk*^*−/−*^ IL17A-GFP mice were exposed to SR as in Fig. 1, and frozen lung sections were analyzed for IL17A-GFP by fluorescence microscopy, Blue = DAPI staining, Green = IL17A-GFP^+^ cells indicated by white arrows. **b** WT IL17A-GFP mice were exposed to SR as in Fig. 1, and lung cells that are GFP^+^ were gated (i.e., GFP^+^ > cell type) and analyzed for numbers of αβ (filled circles), γδ (filled boxes) T cells and neutrophils (filled diamonds). (*n* = 3/group). **c** Proportion of GFP^+^ lung cells (i.e., GFP^+^ > cell type) from SR-exposed WT (filled symbols) or *Itk*^*−/−*^ (open symbols) IL17A-GFP mice that are αβ (circles), γδ (squares) T cells and neutrophils (diamonds) (*n* = 3/group). **d** WT (filled symbols) or *Itk*^*−/−*^ (open symbols) IL17A-GFP mice were exposed to SR as in Fig. 1, and αβ (circles) and γδ (squares) T cells were analyzed for the proportion that is IL17A-GFP^+^ (i.e., cell type > IL17A-GFP^+^) (*n* = 3–4/group). **e** Lung CD4^+^ (circles) and CD8^+^ αβ (triangles) T cells were analyzed for proportion IL17A-GFP^+^ (i.e., cell type > IL17A-GFP^+^) (*n* = 3–4/group).

We next used IL17A-GFP reporter mice to determine the identity of cells producing IL17A during the development of SR-HP. The use of IL17A-GFP reporter mice allows the identification of IL17A producing cells in vivo during the development of disease without resorting to ex vivo stimulation to identify cells, which may misidentify IL17A producers in vivo. Fluorescence microscopic analysis of the lungs of WT IL17A-GFP mice revealed the presence of GFP^+^ cells following SR exposure (see Fig. 2a). Using such IL17A-GFP reporter mice, we analyzed the total number of IL17A^+^ cells by gating first on IL17A-GFP^+^ cells followed by an analysis of cell type in the lungs of WT IL17A-GFP mice (see Supplementary Fig. 2a for example of gating strategy). This numerical analysis revealed that the response in WT mice was dominated by IL17A producing αβ T cells (Fig. 2b).

Analysis of the lungs of SR exposed WT and *Itk*^*−/−*^ IL17A-GFP mice for the proportion of IL17A producing cells (again, by gating first on IL17A-GFP^+^ cells followed by an analysis of cell type in the lungs of IL17A-GFP mice) revealed that a high proportion of γδ T cells produce this cytokine early in the response, followed by αβ T cells in WT mice (Fig. 2c). By contrast, we found that unlike the response in WT mice, which was initially dominated by the IL17A producing γδ T cells early in the response that subsided by 3 weeks, the response in *Itk*^*−/−*^ mice was not, with lower production of IL17A by γδ T cells (Fig. 2c). These results suggest that ITK is critical for the ability of γδ T cells, but perhaps not CD4^+^ T cells, to produce IL17A. Simonian et al. have previously shown that while γδ T cells can contribute to IL17A during SR-induced HP, they are not required for the development of lung inflammation^27^. Indeed, the total number of WT γδ T cells producing IL17A was much lower than WT αβ T cells in the lung (see Fig. 2b). Furthermore, while there was a difference in the ability of *Itk*^*−/−*^ γδ T cells to produce IL17A, this was not critical since mice lacking γδ T cells (*TCRδ*^*−/−*^) or both γδ T cells and ITK (*TCRδ*/*Itk* DKO) exhibited no difference in CD4^+^ T cell production of IL17A following exposure to SR for three weeks (Supplementary Fig. 3a). Thus, while ITK is required for the production of IL17A by γδ T cells, this is not required for the production of IL17A by CD4^+^ T cells in response to SR exposure. We confirmed that ITK plays a critical role in Th17 differentiation by differentiating sort purified naïve CD4^+^ WT and *Itk*^*−/−*^ T cells in vitro under Th17 conditions, as we and others have previously reported (Supplementary Fig. 3d)^11,22–24,31^. Taken together, our data indicate that ITK is required for γδ T cells to produce IL17A during the development of the response to SR in the lung. However, contrary to expectations, ITK is not required to generate an IL17A/Th17 response in the lung in response to SR exposure.

**Fig. 3:**
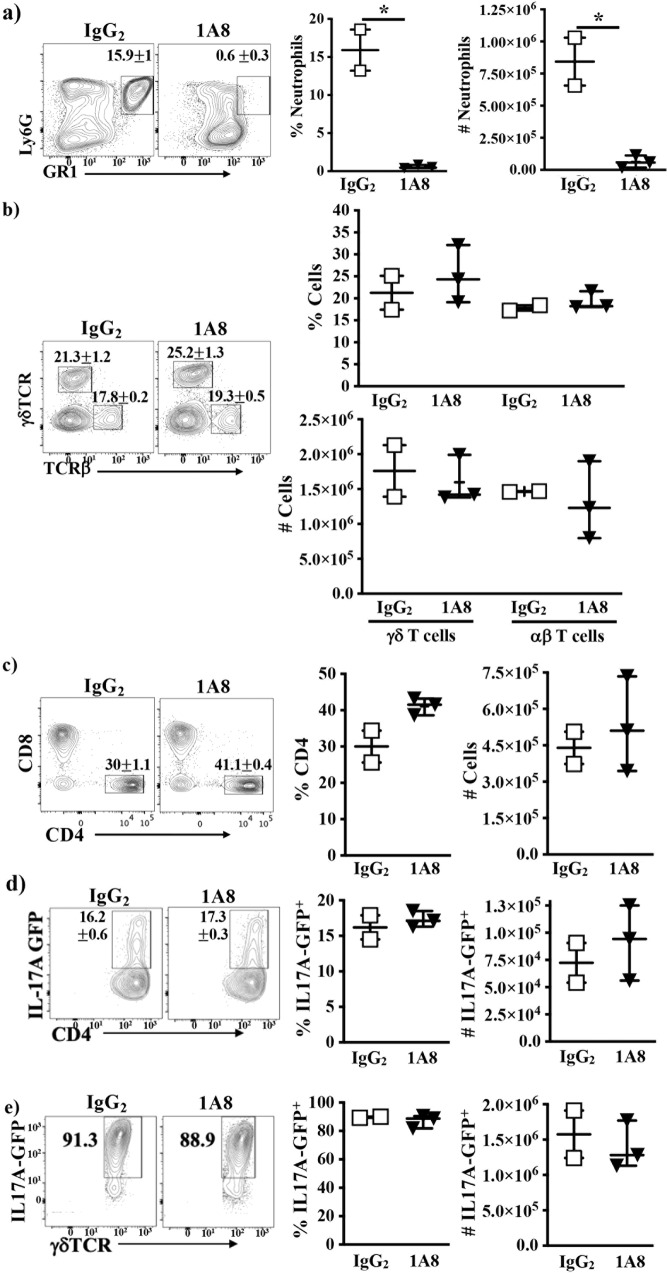
Neutrophils are not required for the recruitment of T cells or their production of IL17A during the development of SR-induced hypersensitivity pneumonitis. WT IL17A-GFP mice were injected with anti-Ly6G (1A8) or rat IgG_2a_ prior to the first SR exposure, then subsequently every other day for 14 days in concert with SR exposure. Flow cytometric analysis of **a** neutrophils (left panel) and proportion and number of neutrophils determined (right panels) (*n* = 2 for IgG_2a_ group, open boxes; *n* = 3 for 1A8 group, filled triangles, *p = 0.005 for proportion and *p = 0.01 for number of neutrophils between IgG_2a_ and 1A8). **b** αβ and γδ T cells, **c** CD4^+^ T cells. **d**, **e** Flow cytometric analysis of IL17A-GFP expression in **d** αβ CD4^+^ cells, **e** γδ T cells (*n* = 2 for IgG_2a_ group, open boxes; *n* = 3 for 1A8 group, filled triangles).

We also found that neutrophils are not significant producers of IL17A (see Fig. 2b, c. Note that there is very little production of IL17A by these cells, and so the line is not detectable on the graph), nor are they required for the development of lung inflammation (Supplementary Fig. 4). Neutrophils are also not required for the presence of αβ T cells or IL17A producing cells, since depletion of neutrophils in WT mice during exposure to SR for two weeks did not affect recruitment of γδ T cells or αβ (including CD4^+^ or CD8^+^ αβ T cells) to the lung (Fig. 3a–c), or the proportion of CD4^+^ αβ T cells, or γδ T cells that produce IL17A in vivo to the lung (Fig. 3d–f). Interestingly, this increase in IL17A producing T cells were mainly compartmentalized in the lung, since similar increases in IL17A producing cells were not found in the spleen or lymph nodes of exposed mice (Supplementary Fig. 3b, c). Taken together, our data suggest that γδ T cells, followed by CD4^+^ αβ T cells are the dominant sources of endogenous IL17A in SR induced HP, and that neutrophils are not significant contributors, nor do they affect the ability of T cells to produce IL17A.

### Enhanced TcR signaling in CD4^+^ T cells the absence of ITK during SR exposure

**Fig. 4:**
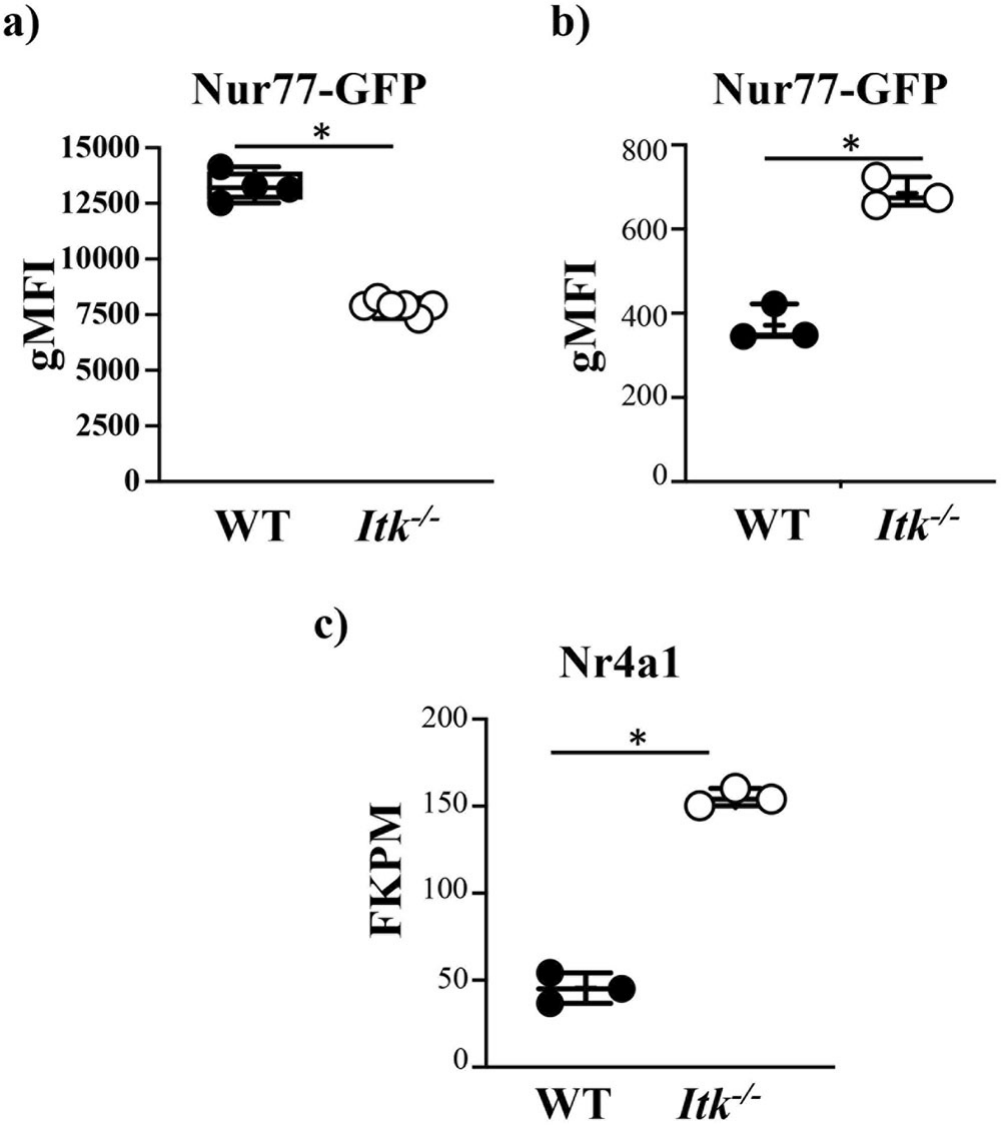
*Itk*^*−/−*^ T cells receive strong signals in vivo during the development of SR-induced hypersensitivity pneumonitis. **a** CD4^+^ T cells from non-exposed WT (filled circles) or *Itk*^*−/−*^ (open circles) Nurr77-GFP mice were analyzed for expression of Nurr77-GFP and MFI plotted (*n* = 4 for WT group; *n* = 6 for *Itk*^*−/−*^ group, *p = 1.1e−7). **b** WT (filled circles) or *Itk*^*−/−*^ (open circles) Nurr77-GFP mice were exposed to SR as in Fig. 1, and lung CD4^+^ T cells were analyzed for expression of Nurr77-GFP and MFI plotted (*n* = 3/group, *p = 0.0006). **c** Expression of *Nr4a1* (gene for Nurr77) determined from RNA-sequencing (FKPM) (data from cells used for RNA-sequencing analysis depicted in Fig. 5, WT (filled circles) or *Itk*^*−/−*^ (open circles), *n* = 3/group, *p = 4.8e−5).

**Fig. 5:**
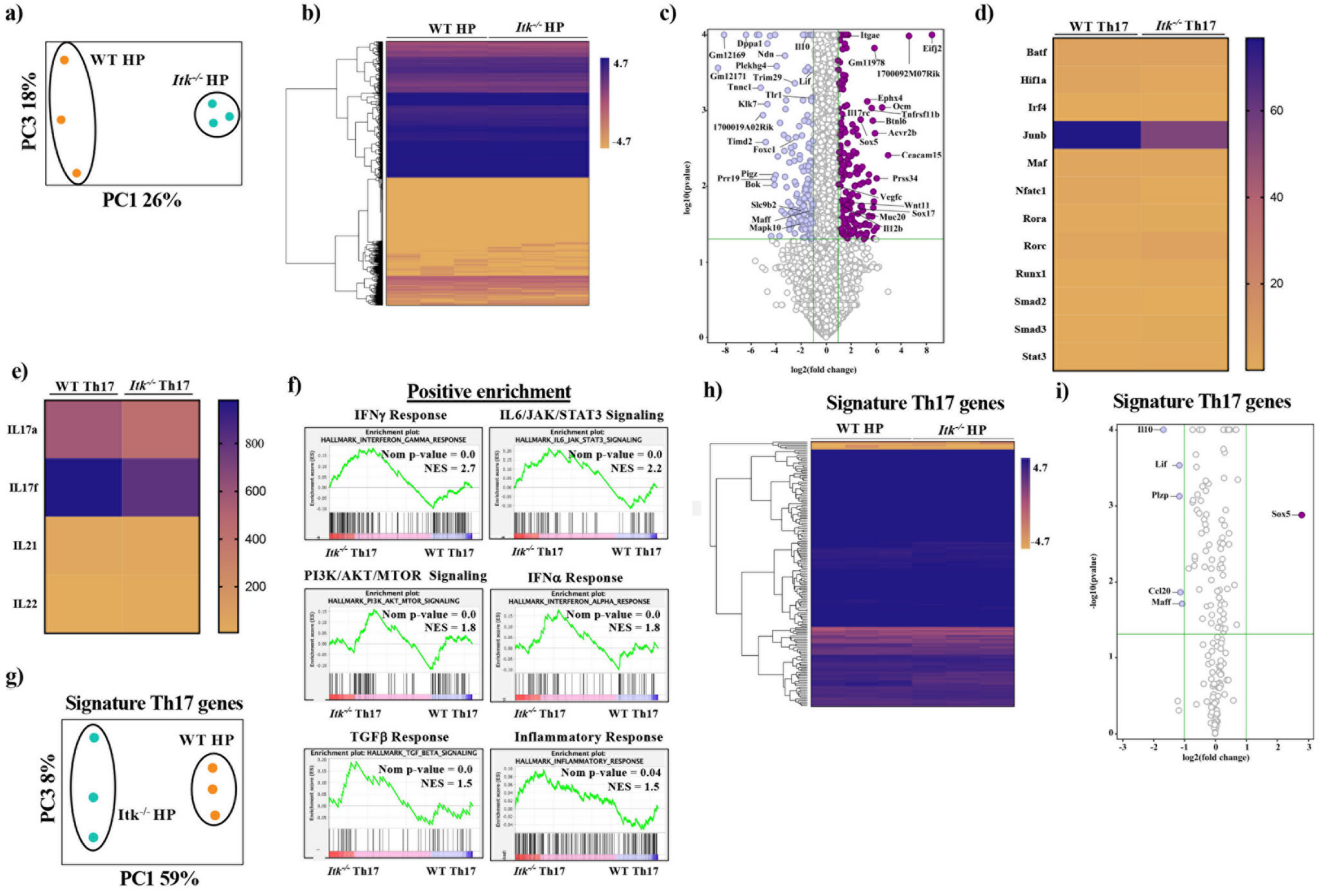
Transcriptomic analysis of SR-induced Th17 cells. WT or *Itk*^*−/−*^ IL17A-GFP/Foxp3-RFP mice were exposed to SR as in Fig. 1 and lung IL17A-GFP^+^/Foxp3^-^/CD4^+^ αβ T cells sort purified, and RNA sequenced. **a** PCA plot of the transcriptome of SR-induced WT (orange circles) or *Itk*^*−/−*^ (green circles) Th17 cells. Axes show the principal components with the greatest difference (PC1 vs. PC3). **b** Heat map and hierarchical clustering of the transcriptome of SR-induced WT or *Itk*^*−/−*^ Th17 cells. **c** Volcano plot of transcripts that are significantly different (>2-fold) between SR-induced WT or *Itk*^*−/−*^ Th17 cells. **d** Heat map of Th17 related transcription factor expression between SR-induced WT or *Itk*^*−/−*^ Th17 cells. **e** Heat map of Th17 associated cytokine expression between SR-induced WT or *Itk*^*−/−*^ Th17 cells GSEA plots of pathways that are significantly positively or negatively enriched between SR-induced WT or *Itk*^*−/−*^ Th17 cells. **f** GSEA plots of pathways that are significantly positively or negatively enriched between SR-induced WT or *Itk*^*−/−*^ Th17 cells. **g** PCA plot of the transcriptome of SR-induced WT(orange circles) or *Itk*^*−/−*^ (green circles) Th17 cells using custom signature Th17 gene set. Axes show the principal components with the greatest difference (PC1 vs. PC3). **h** Heat map and hierarchical clustering of the transcriptome of SR-induced WT or *Itk*^*−/−*^ Th17 cells using custom signature Th17 gene set. **i**) Volcano plot of using custom Th17 gene set transcripts that are significantly different (>2-fold) between SR-induced WT or *Itk*^*−/−*^ Th17 cells.

The surprising finding that the absence of ITK does not affect the ability of SR exposure to generate an IL17A/Th17 response suggests that there are signals received by these cells in vivo, that may overcome the defect observed in vitro or under other conditions in vivo. ITK regulates the strength of the signal from the TcR^17,32^, and so we evaluated whether *Itk*^*−/−*^ T cells receive adequate TcR signals in vivo during exposure to SR by utilizing Nur77-GFP mice^33^. We first examined the strength of TcR signals received by *Itk*^*−/−*^ T cells in vivo by examining Nurr77 expression in T cells from non-exposed mice. These experiments confirm that the absence of ITK results in reduced TcR signals in CD4^+^ T cells (WT 1.7-fold higher Nurr77-GFP expression compared to *Itk*^*−/−*^, Fig. 4a). Next, we examined the receipt of TcR signals in these T cells by inducing SR-HP in WT or *Itk*^*−/−*^ Nur77-GFP mice. WT or *Itk*^*−/−*^ Nur77-GFP mice were exposed intranasally to PBS or SR over the course of two weeks, and Nur77-GFP expression was determined in CD4^+^ T cells in the lungs. We found SR exposure led to significantly more Nur77-GFP expression in *Itk*^*−/−*^ CD4^+^ T cells compared to WT CD4^+^ cells, suggesting that *Itk*^*−/−*^ CD4^+^ T cells receive more TcR signal during SR exposure (*Itk*^*−/−*^ 1.8-fold higher Nurr77-GFP expression compared to WT, Fig. 4b. Higher expression of endogenous Nurr77 in *Itk*^*−/−*^ Th17 cells was confirmed by RNA-sequencing (Fig. 4c, see Fig. 5)). Furthermore, flow cytometric analysis of the expression of Rorγt expression in WT or *Itk*^*−/−*^ Th17 cells isolated from lungs exposed to *SR* for 3 weeks showed no difference in expression of Rorγt (see Supplementary Fig. 5). These signals in vivo may enhance the ability of *Itk*^*−/−*^ T cells to become Th17 cells under these conditions.

### Transcriptome analysis of SR induced WT and *Itk*^*−/−*^ Th17 cells

Our surprise finding that ITK is not required to generate an IL17A/Th17 response in the lung in response to SR exposure led us to explore the transcriptome of the resultant Th17 cells. To do this we sort purified lung Th17 cells from WT and *Itk*^*−/−*^ IL17A-GFP/Foxp3-RFP mice that had been exposed to SR for 3 weeks (CD4^+^IL17A-GFP^+^/Foxp3-RFP^−^), and performed RNA sequencing for comparison of the transcriptome. Principal component analysis (PCA) indicated a clear difference between the two genotypes (Fig. 5a). Hierarchical clustering and heatmap analysis of genes highlighted the small differences between SR-induced WT and *Itk*^*−/−*^ Th17 cells (Fig. 5b). Statistical analysis revealed that less than 2% of genes were significantly different; 197 were significantly upregulated and 172 were downregulated (an unpaired *t*-test [*Itk*^*−/−*^] vs. [WT] *P* ≦ 0.05 FC ≧ 2.0). Visualizing the genes that were significantly different using a volcano plot illustrated these differences (Fig. 5c). Panther pathway analysis^34,35^ of significantly enriched genes revealed that most of the genes that were differentially upregulated genes in the absence of ITK were genes involved in inflammation-mediated by chemokine and cytokine signaling. And most downregulated genes were related to FGF signaling pathway (Supplementary Fig. 6). Analysis of transcripts for Th17 associated transcription factors *Rorc*, *Rora4*, *Stat3*, *Irf4*, *Nfat*, *Runx1*, *Hif1a*, *Batf*, *Junb*, *Smad*, and *cMaf* revealed that only *Junb* expression was reduced in *Itk*^*−/−*^ T cells (Fig. 5d). However, it was notable that transcripts for IL17A and IL17F were significantly decreased in *Itk*^*−/−*^ Th17 cells compared to WT Th17 cells (Fig. 5e). These data suggest that in response to SR in vivo, there is a small percentage of genes that are differentially expressed between WT and *Itk*^*−/−*^ Th17 cells, *Itk*^*−/−*^ Th17 are able to develop, albeit with significantly reduced expression of IL17A.

We next used gene set enrichment assays (GSEA) to derive a better understanding of pathways in enriched in the whole transcriptome of *Itk*^*−/−*^ Th17 cells. This analysis revealed genes involved in the IFNγ response, IL6/JAK/SAT3 signaling, PI3K/AKT/MTOR signaling, IFNα responses, and TGFβ signaling, along with those reduced by KRAS signaling (Fig. 5f, Supplementary Fig. 7) as significantly positively enriched in *Itk*^*−/−*^ Th17 cells. By contrast, genes significantly negatively enriched included those regulated by E2F, MYC, MTORC1 signaling among others (Fig. 5f, Supplementary Fig. 7). This GSEA suggests that inflammatory signals in vivo may provide *Itk*^*−/−*^ cells with signals to rescue the development of Th17 cells during exposure to SR to induce the production of IL17A.

We next analyzed genes that have been previously shown^36^ to be important for Th17 differentiation and function in an attempt to compare the SR-induced Th17 cells that develop in the WT and *Itk*^*−/−*^ mice to these gene sets. We extracted Th17 associated gene sets from Ciofani et al.^36^ and compared them via PCA. Based on analysis of these select Th17 related genes, the results indicated that the *Itk*^*−/−*^ Th17 cells exhibited a slightly different pattern compared to WT Th17 cells (Fig. 5g,h). Volcano plots show more Th17 related genes are significantly downregulated in *Itk*^*−/−*^ Th17 cells (*Il10*, *Lif*, *Plzp*, *Ccl20*, *Maff*), with *Sox5* the only Th17-related gene that is significantly upregulated *Itk*^*−/−*^ Th17 cells (Fig. 5i). These data suggest that WT and *Itk*^*−/−*^ Th17 cells that develop in response to SR are closely related with regard to canonical Th17 transcripts.

Th17 cells can be categorized as non-pathogenic or pathogenic, and we next analyzed the gene sets for genes involved in distinguishing these two types. We compared datasets representing the transcriptome of in vitro generated Th17 cells, i.e., WT naïve CD4^+^ T cells stimulated in the presence of TGFβ and IL6 (considered non-pathogenic), or stimulated in the presence of IL1β, IL6, and IL23 (considered pathogenic)^37^ with SR-induced WT and *Itk*^*−/−*^ Th17 cells by PCA. We found that SR-induced in vivo generated WT or *Itk*^*−/−*^ Th17 cells were more similar to each other, and less similar to the in vitro-generated WT Th17 cells regardless of their manner of generation and potential pathogenicity. This data suggests that SR in vivo-derived Th17 cells may differ from in vitro generated counterparts (Supplementary Fig. 8).

We have previously shown that the absence of ITK reduces the development of Th17 cells during the development of allergic asthma^24^. We, therefore, compared datasets of the transcriptomes of HDM-induced allergic airway inflammation induced Th17 cells with the SR-HP induced WT and *Itk*^*−/−*^ Th17 cells. PCA of the transcriptomes of the three conditions revealed SR-HP-induced Th17 cells are dissimilar to HDM-induced Th17 cells (Supplementary Fig. 9). Interestingly, when we added IL1-β/IL6/IL23 and TGFβ/IL6 induced Th17 cells for comparison with our data set, HDM Th17 cells clustered separately. However, when compared to the Th17 related gene set, all Th17 samples clustered together with the exception of Th17 cells collected from PBS exposed mice (Supplementary Fig. 9). These data suggest that based on their transcriptomes different types of Th17 cells are fairly similar, but SR-induced Th17 cells can be distinguished based on their transcriptomes.

### Chromatin accessibility of SR induced WT or *Itk*^*−/−*^ Th17 cells

**Fig. 6:**
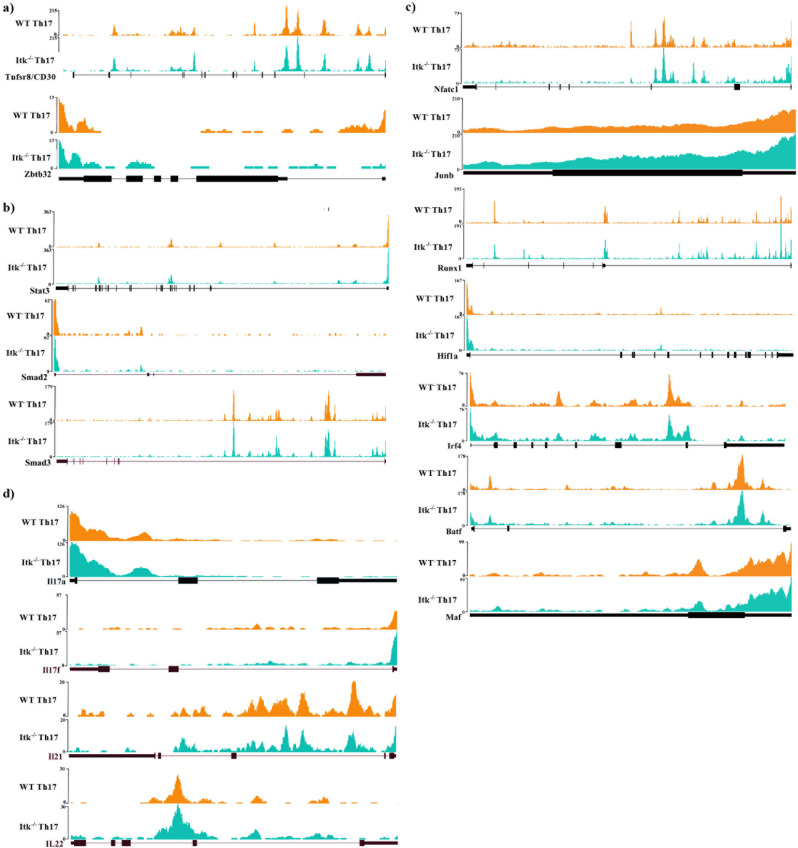
ATAC-Seq analysis of SR-induced Th17 cells. WT (orange tracks) or *Itk*^*−/−*^ (green tracks) IL17A-GFP mice were exposed to SR as in Fig. 1 and lung IL17A-GFP^+^/Foxp3-RFP^-^/CD4^+^ αβ T cells sort purified, and processed as described for ATAC-Seq. **a** Tracks of *Tnfr8* and *Zbt32* loci from WT or *Itk*^*−/−*^ Th17 cells. **b** Tracks of the loci of IL6 and TGFβ related transcription factors *Stat3*, *Smad2*, and *Smad3*. **c** Tracks of the loci of Th17 related transcriptional factors *Nfatc1*, *Junb*, *Runx1*, *Hif1a*, *Irf4*, *Batf*, and *Maf*. **d** Tracks of the loci of Th17 related cytokines *Il17a*, *Il17f*, *Il21*, and *Il22*.

To further explore the ability of *Itk*^*−/−*^ T cells to become Th17 cells in response to SR, we performed ATAC-Seq to examine the accessibility of the nuclear chromatin. Purified lung Th17 cells from WT and *Itk*^*−/−*^ IL17A-GFP/Foxp3-RFP mice that had been exposed to SR for 3 weeks (CD4^+^IL17A-GFP^+^/Foxp3-RFP^−^) were collected and prepared for ATAC-Seq. We found 60 significantly different peaks (*p* = 1.43e−9). However, with the exception of *Zbtb32* we did not find many differences that matched the differences observed by RNA sequencing. Peaks near *Tnfrsf8* and *Zbtb32* were significantly lower in *Itk*^*−/−*^ Th17 cells (Fig. 6a), although notably, there was no difference in chromatin accessibility near canonical Th17 transcription factors and cytokines, including IL17A (Fig. 6b–d). Taken together, this analysis of the transcriptome and chromatin accessibility of WT and *Itk*^*−/−*^ Th17 cells support the conclusion that SR induces inflammatory signals that are able to rescue the development of Th17 response in the absence of ITK, and although *Itk*^*−/−*^ Th17 cells produce lower levels of transcripts for IL17A, this is sufficient to result in lung inflammation.

## Discussion

Th17 cells play important roles in the immune response to infection at mucosal surfaces, as well as in the development of a number of diseases including asthma, HP, and autoimmune diseases such as rheumatoid arthritis^1–7^. Although the signaling pathways downstream of the TCR that drives the development of Th17 cells remain unclear, we and others have shown that the TCR-activated tyrosine kinase ITK is critical for the development of these cells^11,24,25^. Here, however, in a mouse model of inflammatory lung disease HP dependent on IL17A, we find that despite its well-documented role in the differentiation of Th17 cells^22,24^, SR-induced inflammation renders Th17 differentiation independent of ITK. These findings have implications for understanding Th17 differentiation programs of IL17A-driven inflammatory diseases.

While CD4^+^ T cells are prominent producers of IL17A^38^, previous studies have suggested that while there are elevated levels of IL17A in the lung in SR-induced HP, there is a surprisingly small percentage of CD4^+^ Th17 cells in the lung, suggesting that IL17A may be produced by other sources as well^27^. γδ T cells are also a well-known source of IL17A in response to antigen and bacterial infection, and other studies have also shown that in *B. subtilis* and Pigeon Droppings Extract induced model of HP, IL17A concentration was significantly increased in the lung and Vγ6Vδ1 or Vγ4 γδ expressing T cells are the predominant population of IL17A producing cells^39,40^. Furthermore, neutrophils, which can regulate Th1 and Th17 responses^41^, have been reported to be the major producers of IL17A in SR-HP^42^. Thus while a variety of cell types have been reported to produce IL17A during SR-HP, the cellular source of IL17A is unclear. A better understanding of how this disease develops will lead to the development of better therapy since mechanisms involved in Th17 driving inflammatory lung disease are far less understood in comparison to the classic Th2 mediated asthma. In this work, we utilized mice able to express GFP when they express IL17A (IL17A-GFP mice). This method allows the determination of IL17A expression in vivo without resorting to ex vivo stimulation, which only displays the potential to express cytokine and can be misleading. Indeed, using ex vivo stimulation in a model of SR-induced HP, Hasan et al., previously reported that neutrophils have the potential to express IL17A during HP and proposed that neutrophils and not T cells are responsible for the increased IL17A^42^. However, our studies using the IL17A-GFP reporter indicate that a large number of αβ CD4^+^ T cells are responsible for this expression and that this was minimally observed in the neutrophil population. These findings support the use of such cytokine reporter mice to more accurately determine the dynamics of cytokine expression in vivo during disease. Hippler et al., have also recently utilized different IL17A reporter mice to show that neutrophils also do not produce IL17A during infection with *Candida albicans*^43^, suggesting that although neutrophils can be producers of IFNγ ^44^, they may not be significant IL17A producers. αβ T cells are recognized as important mediators of pathology in HP, and IL17A has been established as the predominant cytokine in SR-induced HP^27,28,30^. Our results establish that CD4^+^ αβ T cells are the major producers of this IL17A, suggesting that SR-HP develops due to a predominant Th17 response driven by IL17A from CD4^+^ αβ T cells. This IL17A may then recruit neutrophils to the lung to contribute to the disease, but these neutrophils contribute little if any IL17A to the process.

We observed that the proportion and number of γδ T cells increase over the course of SR-HP inflammation and that the loss of ITK signaling leads to a decrease in the proportion and number of these T cells in the lung earlier in the disease, which later recovers to levels comparable to WT. This finding would suggest that ITK plays an important role in the early production of IL17A by conventional γδ T cells during the development of SR-HP. Regardless, based on the numbers of IL17A producing T cells, γδ T cells make a minor contribution compared to CD4^+^ αβ T cells.

We and others have documented a significant role for ITK in the differentiation of Th17 cells, including in response to multiple cytokines, including IL6/TGFβ, IL21/TGFβ, IL1/IL6, IL1/TGFβ, and IL6/IL23/ TGFβ^22,24^. We therefore initially predicted that the absence of ITK would lead to a reduction in response in SR-induced HP. However, this was not the case, since the absence of ITK did not lead to any alteration in inflammation in the lung tissue, recruitment of αβ or γδ T cells to the lung (although delayed in the case of γδ T cells). Importantly there was no effect on the ability of CD4^+^ αβ T cells to produce IL17A.

Analysis of receipt of TcR signals downstream of ITK using Nurr77-GFP reporter mice suggests that *Itk*^*−/−*^ T cells receive strong signals during SR exposure which may rescue Th17 development. Indeed, transcriptomic analysis of SR induced WT and *Itk*^*−/−*^ Th17 cells by GSEA revealed that these cells are very similar, however, there was enhanced expression of genes involved in inflammation and in metabolism in *Itk*^*−/−*^ Th17 cells. Metabolic pathways downstream of ITK, such as mTOR signaling regulate T cell lineage commitment and functions. Both mTORC1 and mTORC2 are needed for Th17 cell differentiation and their production of IL17A is positively regulated by mTORC1^45,46^. Furthermore, Th17 cells require fatty acid synthesis^47,48^, and so the reduced expression of genes involved in fatty acid metabolism in the absence of ITK may allow these cells more access to these critical nutrients for their differentiation and function. These data suggest there is a small set of genes and pathways that positively regulate Th17 cells concurrent with metabolic changes that may rescue ITK signaling, although transcripts of *Il17a* were significantly reduced in the *Itk*^*−/−*^ Th17 cells. Interestingly, Gaublomme et al., reported that *Gpr65*, *Plzp*, *Toso*, and *Cd5l* are identifiers of Th17 pathogenicity, yet we found that only *Plzp* was significantly downregulated in *Itk*^*−/−*^ Th17 cells. Chromatin accessibility analysis by ATAC-Seq also revealed high similarity between WT and *Itk*^*−/−*^ Th17 cells including in canonical Th17 genes.

The finding that Th17 cells develop in SR exposed mice in the absence of ITK supports the view of plasticity of Th17 cells that is dependent on their environment^49^. Indeed, the difference in the transcriptomes of Th17 cells from HDM exposed mice and SR exposed mice is also likely explained by this. Given our previous work indicates that ITK is required for the development of Th17 cells in vitro, and in an ovalbumin-induced model of allergic asthma^24^, our findings suggest that the role of ITK is more complicated and nuanced in Th17 development. Indeed, SR has a number of PAMPS that may induce a very different environment in the lung than that induced during ovalbumin-induced allergic asthma^50^. This different environment may overcome the signaling requirement for ITK in the development of Th17 cells. However, our work suggests that the transcriptome of the resulting SR-induced Th17 cells are quite similar, although we should note that the absence of ITK does affect their ability to produce IL17A, since the *Itk*^*−/−*^ Th17 cells express fewer transcripts for IL17A, suggesting that ITK’s role is blunted in response to SR exposure.

In conclusion, while we and others have previously shown that ITK is required for the development of conventional Th17 cells)^11,22–24,31^, our findings here suggest that the requirement for ITK in T cell production of IL17A is complex, and may depend on the conditions in vivo. These findings have important implications for the understanding of the signaling pathways that are required for the development of Th17 cells. They also have implications for the development of therapeutic approaches to inhibit IL17 driven inflammatory diseases, since inhibitors targeting ITK may have utility for some diseases where Th17 cells have a role, there may be other diseases, such as SR-driven HP, where they may have less utility.

## Methods

### Mice

All mice were on a C57Bl/6 background. *Itk*^*−/−*^, TCRδ (γδ T cell-deficient mice, B6.129P2-*Tcrd*^*tm1Mom*^/J, Jax Labs), *IL4Rα*^*−/−*^ (a kind gift from Dr. Frank Brombacher, University of Cape Town, South Africa, via Dr. Fred Finkelman, University of Cincinnati^51^). IL17A-GFP reporter mice (C57BL/6-*Il17a*^*tm1Bcgen*^/J were purchased from Biocytogen, and Foxp3-RFP reporter mice (C57BL/6-*Foxp3*^*tm1Flv*^/J) were from the Jackson Laboratory (Bar Harbor, ME). Reporter strains were crossed to generate IL17A-GFP/Foxp3-RFP dual reporter strains in WT or *Itk*^*−/−*^ background^52^. TCRδ/*Itk*^*−/−*^ were previously described ^53^. All mice were housed in a specific pathogen-free environment, and male and female mice were between 6 and 8 weeks of age when used. All experiments were approved by the Office of Research Protection’s Institutional Animal Care and Use Committee at The Pennsylvania State University and Cornell University.

### SR-induced HP

Mice were exposed intranasally to 150 μg extract of SR (obtained by American Type Tissue Collection catalog no. 153347) 3 times a week up to 3 weeks^28^. Twenty-four hours after the last exposure, mice were sacrificed and analyzed, and measurements were taken from distinct samples.

### Neutrophil Depletion

Neutrophils were depleted as previously described^54^. Briefly, WT IL17A-GFP/Foxp3-RFP mice were injected intravenously with 200 μg of 1A8 (BioXcell) or rat IgG2a (BioXcell) in 100 μl PBS prior to the first SR exposure, then subsequently every other day for 14 days in concert with SR induced HP.

### Cell isolation, flow cytometry analysis, and T cell stimulation

Cells were isolated from BAL, lungs, spleen, and draining lymph nodes and analyzed by flow cytometry as previously described^55^. Cells were stained with the following antibodies at the concentration of 1:200: CD16/32 (i.e., Fc block, eBioscience), Fixable Viability Dye eFluor 506, anti-Ly6G PE-Cy7 or eFluor 450, anti-CD117 FITC, SiglecF PE, CD11b PE-Texas Red, anti-CD11c APC, anti-MHC II AF700, anti-CD49b PerCP eF710, anti-FcεRIα PECy7, anti-F4/80 APC/Cy7, anti-IL17A PerCP-CY5.5 (Ebioscience), anti-TCRβ APC-CY7 (Biolegend), anti-CD4 Alexa Fluor 700 or eFluor 450, anti-CD8α PE-Texas Red or PerCP-Cy5.5, anti-TCRδ APC, anti-NK1.1 Allophycocyanin, anti-CD44 V500, anti-CD62L PE-Cy7, anti-CD11b Alexa Fluor 647 (BD Pharmingen), anti-B220 Alexa Fluor 700, PE-PBS57 loaded CD1d tetramer was from the National Institute of Allergy and Infectious Diseases Tetramer Facility. Purified anti-CD3 and CD28 antibodies were from BD Biosciences. In some cases, cells were stimulated with PMA and Ionomycin followed by an analysis of intracellular cytokine as previously described^56^. Cells were analyzed using a BD FACS Aria II flow cytometer and analyzed with FlowJo software.

### RNA sequencing and analysis

WT or *Itk*^*−/−*^ IL17A-GFP/Foxp3-RFP mice were exposed to SR for up to 3 weeks and CD4^+^ IL17A-GFP^+^Foxp3-RFP^−^ cells from SR exposed lungs were sorted using BD FACSAria Fusion Fluorescence Activated Cell Sorter. Isolated RNA from sorted cells was sequenced by the RNA Sequencing Core (Cornell University, College of Veterinary Medicine). Data were analyzed using Ingenuity Pathway Analysis (IPA; QIAGEN Inc., https://www.qiagenbioinformatics.com/products/ingenuitypathway-analysis) or GeneSpring software version 14.1 (Agilent)), and gene set enrichment analysis (GSEA)^57,58^ was used to determine biological pathways of significance. Panther pathway analysis was used to determine pathways related to genes that were significantly different in WT or *Itk*^*−/−*^ Th17 cells^34,35^. Comparison of our transcriptome data with data from in vitro generated Th17 cells cultured (anti-CD3 in the presence of either IL1β, IL6 and IL23 (considered pathogenic, data set GSM3913969) or TGFβ and IL6 (considered non-pathogenic, GSM3913966), obtained from NCBI Gene Expression Omnibus database series GSE133641 contributed by Ichiyama et al., 2019). Comparison with HDM-induced allergic airway inflammation induced Th17 cells (GSE100858,^59^). All FPKM values were converted to log2 for data analysis unless otherwise indicated. Excluding micro RNAs, we filtered 20,024 genes, based on the coefficient of valuation (CV) (for at least 1 out of 2 conditions was set to < 50.0 percent), and filtered on the error where the CV was greater than 100.

### ATAC-sequencing and analysis

Sort purified CD4^+^ IL17A-GFP^+^Foxp3-RFP^-^ from lungs of WT or *Itk*^*−/−*^ IL17A-GFP/Foxp3-RFP mice exposed to SR were isolated as previously described. Isolated cells were processed, sequenced, and analyzed^60,61^ by the Transcriptional Regulation & Expression Facility (Cornell University, Center for Reproductive Genomics and College of Veterinary Medicine) and Novogene. BedGraph files were viewed in UCSC genome browser^62^.

### Reverse transcription/quantitative PCR

RNA was extracted either using TRIZOL reagent (Invitrogen) or an RNAeasy kit (Invitrogen) and cDNA was generated using a You Prime First-Strand beads kit (GE Healthcare). qPCR was then performed using a 7500 Fast Real-Time PCR instrument (Applied Biosystems). Data were analyzed using the comparative threshold cycle 2^−ΔΔCT^ method, normalized to respective and the values were expressed as fold change compared to WT mice.

### Histopathological analysis of lung sections

Lung samples were fixed in 4% paraformaldehyde followed by staining with H&E^63^. Histological samples were analyzed and the severity of pathology was blindly assessed.

### Statistics and reproducibility

Student’s *t*-test or two-way ANOVA analysis were performed using GraphPad Prism version 5.00 for Windows (GraphPad, San Diego, CA). Differences with probability *p* ≤ 0.05 were considered statistically significant. Statistical analysis of two groups compared by Student’s *t*-test (unpaired). Statistical analysis of three or more groups was compared using a two-way ANOVA. Biological replicates or sample sizes are indicated in the figure legends.

### Reporting summary

Further information on research design is available in the Nature Research Reporting Summary linked to this article.

## Supplementary information

Supplementary Information

Reporting Summary

## Data Availability

The datasets generated during and/or analyzed during the current study are available in the NCBI GEO repository https://www.ncbi.nlm.nih.gov/geo/query/acc.cgi?acc=GSE1165711. The source data underlying the graph in the paper are deposited in Figshare^64^.
