# Supplementary material for: ITK independent development of Th17 responses during hypersensitivity pneumonitis driven lung inflammation
Source: Commun Biol. 2022 Feb 24;5:162. doi: 10.1038/s42003-022-03109-1 (PMC8873479; doi:10.1038/s42003-022-03109-1)
Supplement: Supplementary file 1 — Supplementary Information [file 42003_2022_3109_MOESM1_ESM.pdf]

Supplementary Materials:

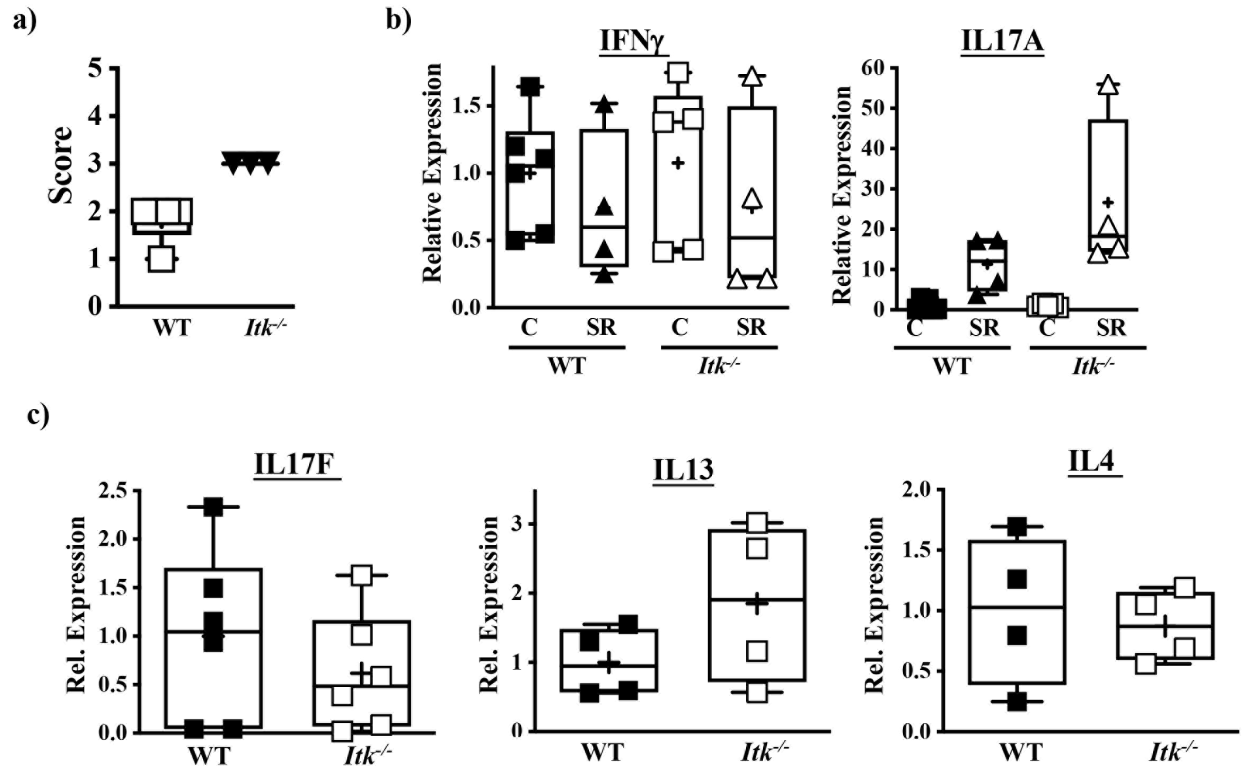

Supplementary Figure 1. Itk does not affect lung inflammation, or the expression of Th1, 2 or Th17 cytokines in the lung during SR-induced Hypersensitivity pneumonitis. a) WT or *Itk*$^{-/-}$ mice were exposed to PBS or SR as in figure 1, lung sections analyzed by histology and scored, or **b**), lung cells collected and analyzed by RT-PCR for expression of IFNγ or IL17A, or **c**) ,IL17F, IL13 or IL4.

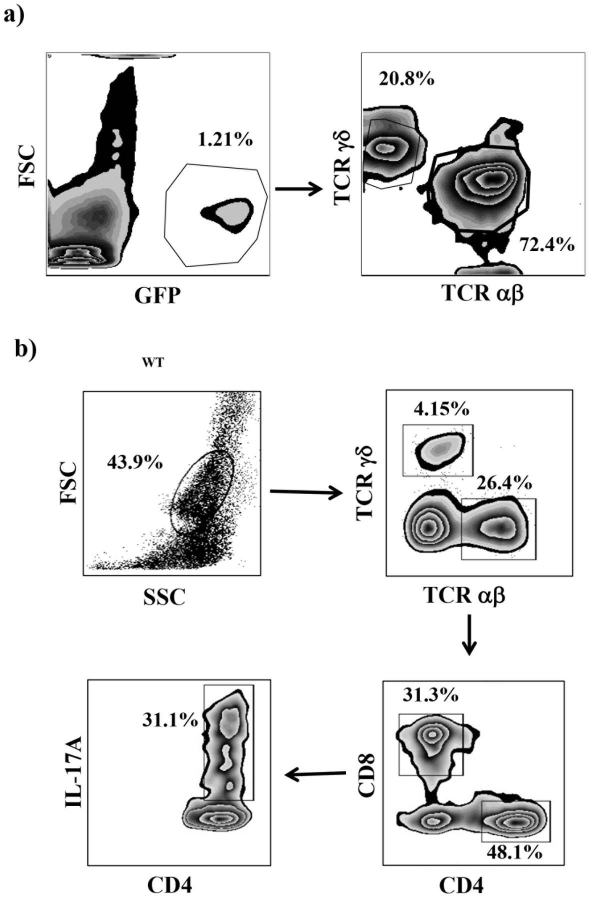

Supplementary Figure 2. Gating for analysis of IL17A-GFP^{+} cells in the lung. a) WT or *Itk*$^{-/-}$ IL-17A-GFP mice were exposed to SR as in figure 1, lung cells collected and analyzed by flow cytometry. Cells were first gated for FSC vs GFP, then TcR αβ vs γδ followed by gating for αβ T cell subsets CD4 and CD8 (not shown). **b**) Cells were first gated for FSC vs SSC, then TcR αβ vs γδ followed by gating for αβ T cell subsets CD4 and CD8, followed by analysis for GFP expression.

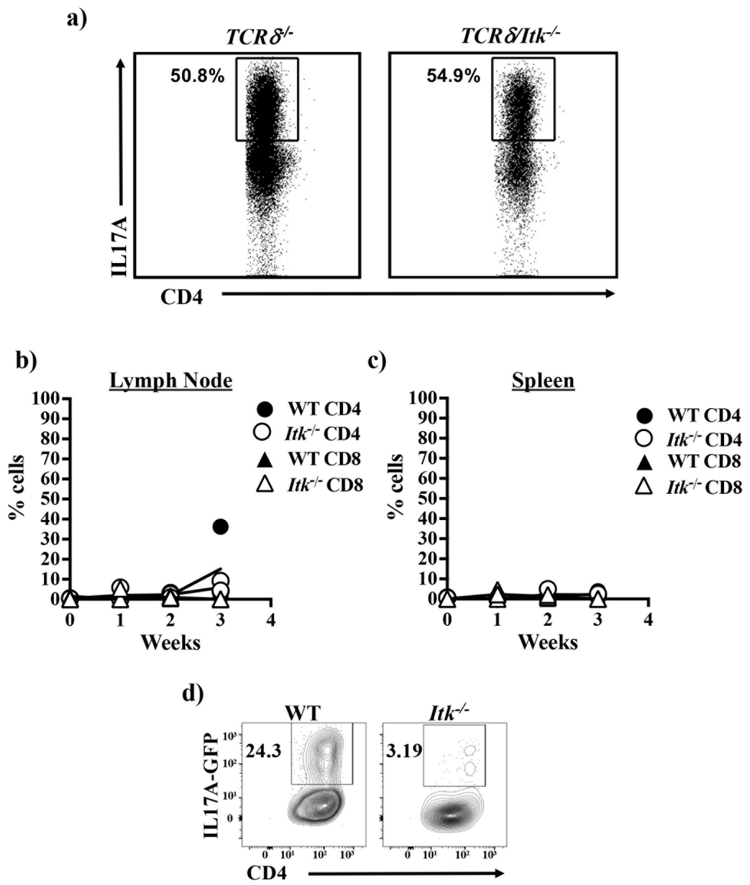

Supplementary Figure 3. γδ T cells do not affect production of IL17A by CD4$^+$ T cells in the presence or absence of Itk. a) *Tcrδ$^{-/-}$* or *Tcrδ/Itk* DKO mice were exposed to SR as in figure 1, and lung cells collected and, restimulated in vitro using PMA/Ionomycin, fixed, and stained for intracellular IL17A, followed by analysis of αβ CD4$^+$ T cells by flow cytometry. **b-c**) WT or *Itk$^{-/-}$* mice were exposed to PBS or SR as in figure 2, and Lymph nodes (**b**) or (**c**) spleen CD4$^+$ and CD8$^+$ αβ T cells were analyzed for proportion IL17A-GFP$^+$ (i.e. cell type>IL17A-GFP$^+$)(n=3/group). **d**) purified naïve CD4$^+$ T cells from WT or *Itk$^{-/-}$* IL17A-GFP mice were stimulated in vitro under Th17 conditions for 3 days, and analyzed for IL17A-GFP expression by flow cytometry.

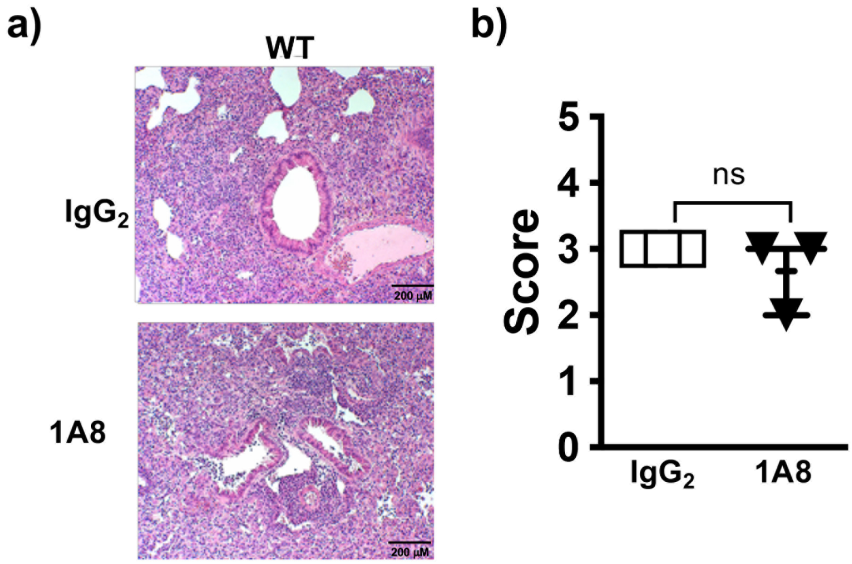

Supplementary Figure 4. Neutrophils are not required for the development of lung inflammation during SR-induced Hypersensitivity pneumonitis. a) WT mice were injected with mAb 1A8 to deplete neutrophils, or IgG_2 (as a control), then exposed to SR as in figure 3. **b**) lung sections analyzed by histology and scored.

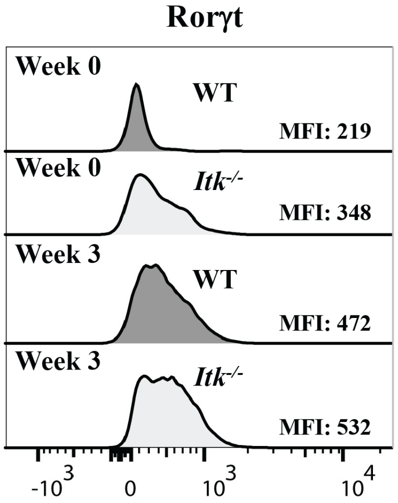

Supplementary Figure 5. No difference in expression of Rorγt in WT and *Itk*$^{-/-}$ Th17 cells during SR-induced Hypersensitivity pneumonitis. Lung IL17A-GFP^{+} CD4^{+} T cells from control WT or *Itk*$^{-/-}$ IL17A-GFP/Foxp3-RFP mice (week 0) or those exposed to SR for 3 week (week 3) were analyzed for expression of Rorγt**.**

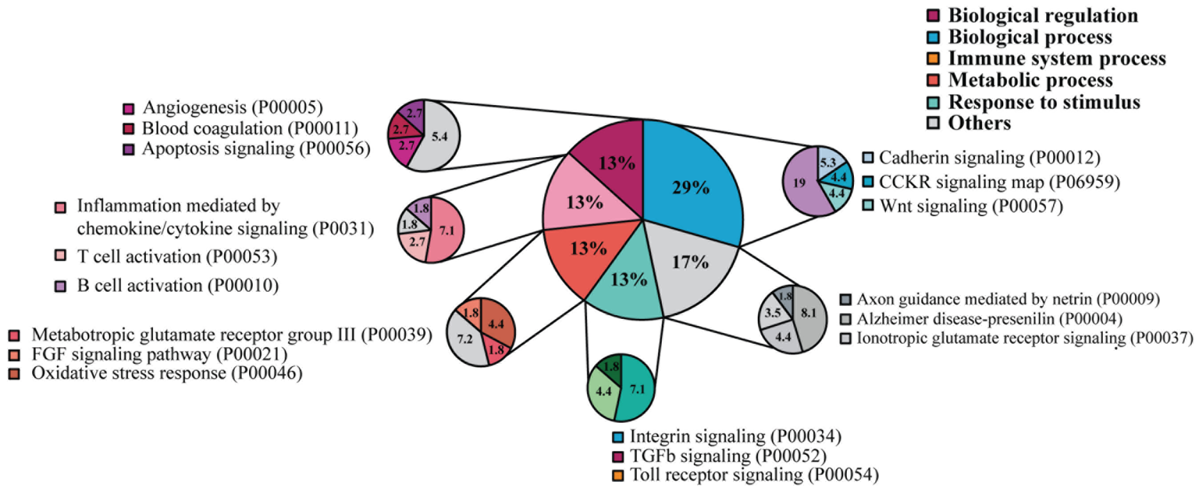

Supplementary Figure 6. Analysis of the biological pathways encoded by the transcriptome of SR-induced WT and *Itk*$^{-/-}$ Th17 cells. WT or *Itk*$^{-/-}$ IL-17A-GFP mice were exposed to SR as in figure 1 and lung IL17A-GFP^{+}/CD4^{+} αβ T cells sort purified, and RNA sequenced. List of biological pathways representing differentially expressed genes between WT or *Itk*$^{-/-}$ Th17 cells.

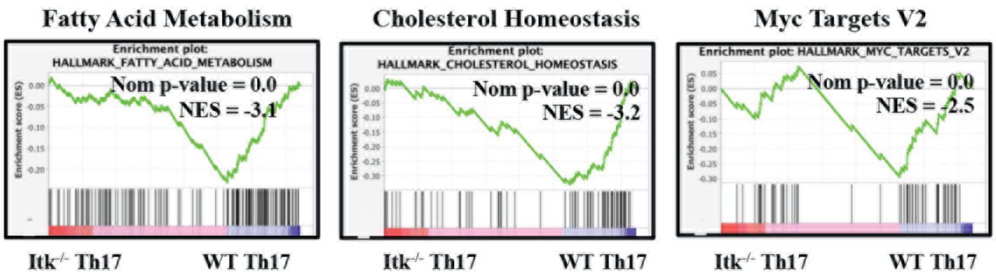

Supplementary Figure 7. Extended GSEA analysis of SR-induced Th17 cells vs other Th17 cells. GSEA plots of pathways that are significantly positively or negatively enriched between SR-induced WT or *Itk*$^{-/-}$ Th17 cells.

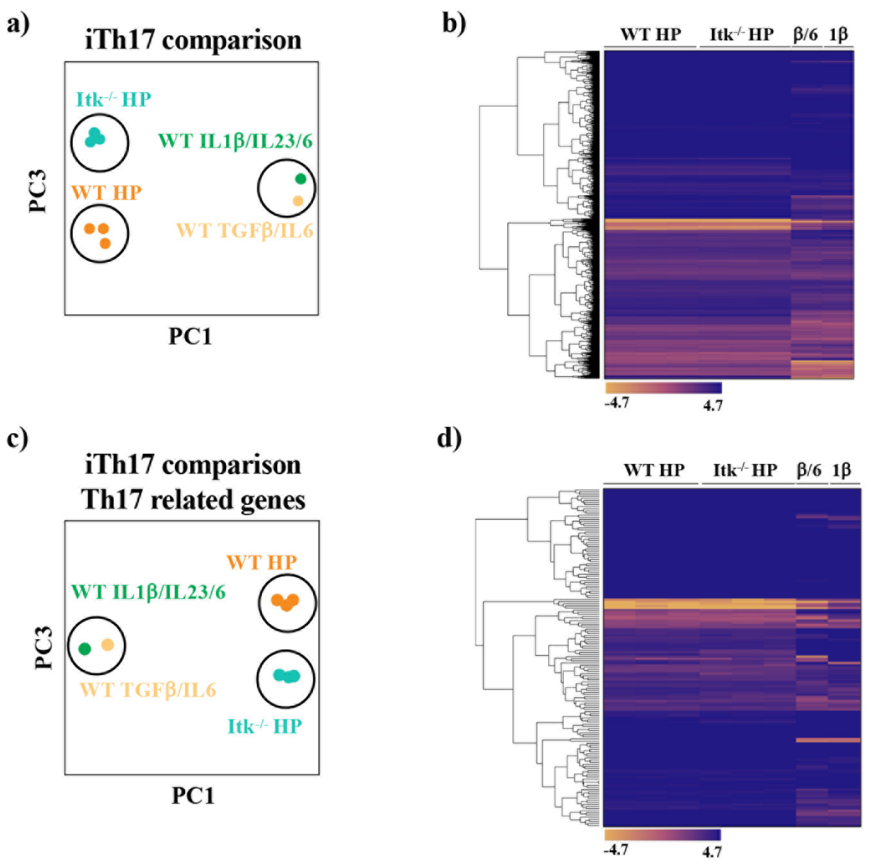

Supplementary Figure 8. Transcriptomic analysis of SR-induced Th17 cells vs other Th17 cells. a) PCA plot of transcriptome of SR-induced WT or *Itk*$^{-/-}$ Th17 cells vs. in vitro derived Th17 cells as indicated. Axes show the principal components with the greatest difference (PC1 vs PC3). **b**) Heat map and hierarchical clustering of transcriptome of SR-induced WT or *Itk*$^{-/-}$ Th17 cells vs. in vitro derived Th17 cells as indicated. **c**) PCA plot of transcriptome of SR-induced WT, *Itk*$^{-/-}$ Th17 cells, and in vitro derived Th17 cells analyzing Th17 related genes. Axes show the principal components with the greatest difference (PC1 vs PC3). **d**) Heat map and hierarchical clustering of transcriptome of SR-induced WT, *Itk*$^{-/-}$ Th17 cells, and in vitro derived Th17 cells analyzing Th17 related genes.

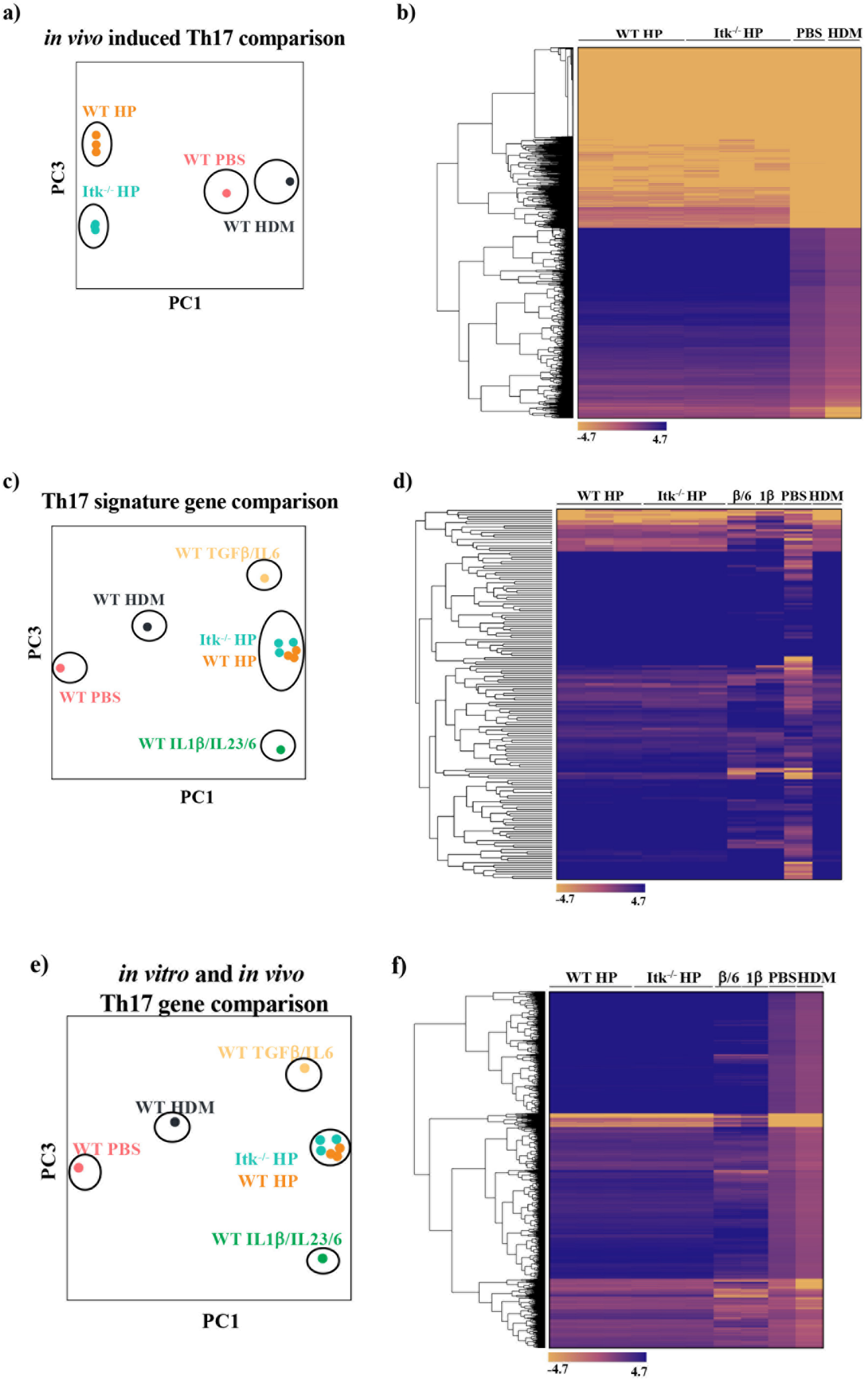
a)
in vivo induced Th17 comparison
WT HP
WT PBS
Itk-/- HP
WT HDM
PC3
PC1
b)
WT HP
Itk-/- HP
PBS
HDM
-4.7
4.7
c)
Th17 signature gene comparison
WT TGFβ/IL6
WT HDM
Itk-/- HP
WT HP
WT PBS
WT IL1β/IL23/6
PC3
PC1
d)
WT HP
Itk-/- HP
β/6
1β
PBS
HDM
-4.7
4.7
e)
in vitro and *in vivo*
Th17 gene comparison
WT TGFβ/IL6
WT HDM
WT PBS
Itk-/- HP
WT HP
WT IL1β/IL23/6
PC3
PC1
f)
WT HP
Itk-/- HP
β/6
1β
PBS
HDM
-4.7
4.7

Supplementary Figure 9. Transcriptomic analysis of SR-induced Th17 cells vs other Th17 cells. **a**) PCA plot of transcriptome of SR-induced WT or *Itk*$^{-/-}$ Th17 cells vs. HDM-induced or PBS-induced WT Th17 cells as indicated. Axes show the principal components with the greatest difference (PC1 vs PC3). **b**) Heat map and hierarchical clustering of transcriptome of SR-induced WT or *Itk*$^{-/-}$ Th17 cells vs. HDM-induced or PBS-induced WT Th17 cells as indicated. **c**) PCA plot of transcriptome of SR-induced WT or *Itk*$^{-/-}$ Th17 cells vs. in vitro derived, HDM or PBS induced Th17 cells as indicated. Axes show the principal components with the greatest difference (PC1 vs PC3). **d**) Heat map and hierarchical clustering of transcriptome of SR-induced WT or *Itk*$^{-/-}$ Th17 cells vs. in vitro derived, HDM or PBS induced Th17 cells as indicated. **e**) PCA plot of transcriptome of SR-induced WT or *Itk*$^{-/-}$ Th17 cells vs. in vitro derived, HDM or PBS induced Th17 cells as indicated analyzing Th17 related genes. Axes show the principal components with the greatest difference (PC1 vs PC3). **f**) Heat map and hierarchical clustering of transcriptome of SR-induced WT or *Itk*$^{-/-}$ Th17 cells vs. in vitro derived, HDM or PBS induced Th17 cells as indicated analyzing Th17 related genes.
