# Supplementary material for: ITK independent development of Th17 responses during hypersensitivity pneumonitis driven lung inflammation
Source: Commun Biol. 2022 Feb 24;5:162. doi: 10.1038/s42003-022-03109-1 (PMC8873479; doi:10.1038/s42003-022-03109-1)
Supplement: Supplementary file 2 — Reporting Summary [file 42003_2022_3109_MOESM2_ESM.pdf]

nature research

Corresponding author(s): Avery August

Last updated by author(s): November 3, 2021

Reporting Summary

Nature Research wishes to improve the reproducibility of the work that we publish. This form provides structure for consistency and transparency in reporting. For further information on Nature Research policies, see our Editorial Policies and the Editorial Policy Checklist.

Statistics

For all statistical analyses, confirm that the following items are present in the figure legend, table legend, main text, or Methods section.

n/a	Confirmed	
☐	☒	The exact sample size (*n*) for each experimental group/condition, given as a discrete number and unit of measurement
☐	☒	A statement on whether measurements were taken from distinct samples or whether the same sample was measured repeatedly
☐	☒	The statistical test(s) used AND whether they are one- or two-sided<br>*Only common tests should be described solely by name; describe more complex techniques in the Methods section.*
☒	☐	A description of all covariates tested
☒	☐	A description of any assumptions or corrections, such as tests of normality and adjustment for multiple comparisons
☒	☐	A full description of the statistical parameters including central tendency (e.g. means) or other basic estimates (e.g. regression coefficient) AND variation (e.g. standard deviation) or associated estimates of uncertainty (e.g. confidence intervals)
☐	☒	For null hypothesis testing, the test statistic (e.g. *F*, *t*, *r*) with confidence intervals, effect sizes, degrees of freedom and *P* value noted<br>*Give P values as exact values whenever suitable.*
☒	☐	For Bayesian analysis, information on the choice of priors and Markov chain Monte Carlo settings
☒	☐	For hierarchical and complex designs, identification of the appropriate level for tests and full reporting of outcomes
☒	☐	Estimates of effect sizes (e.g. Cohen's *d*, Pearson's *r*), indicating how they were calculated

Our web collection on statistics for biologists contains articles on many of the points above.

Software and code

Policy information about availability of computer code

Data collection	*Provide a description of all commercial, open source and custom code used to collect the data in this study, specifying the version used OR state that no software was used.*
Data analysis	FlowJo V9<br>GraphPad Prism V8<br>Ingenuity Pathway Analysis<br>GeneSpring 14.1<br>Panther Pathway Analysis<br>Gene Set Enrichment Analysis

For manuscripts utilizing custom algorithms or software that are central to the research but not yet described in published literature, software must be made available to editors and reviewers. We strongly encourage code deposition in a community repository (e.g. GitHub). See the Nature Research guidelines for submitting code & software for further information.

Data

Policy information about availability of data

All manuscripts must include a data availability statement. This statement should provide the following information, where applicable:

- Accession codes, unique identifiers, or web links for publicly available datasets
- A list of figures that have associated raw data
- A description of any restrictions on data availability

The datasets generated during and/or analyzed during the current study are available from the corresponding authors on reasonable request.

Field-specific reporting

Please select the one below that is the best fit for your research. If you are not sure, read the appropriate sections before making your selection.

☒ Life sciences ☐ Behavioural & social sciences ☐ Ecological, evolutionary & environmental sciences

For a reference copy of the document with all sections, see nature.com/documents/nr-reporting-summary-flat.pdf

Life sciences study design

All studies must disclose on these points even when the disclosure is negative.

Sample size	Samples sizes were initially determined by power analyses. Subsequent samples sizes were determined based on preliminary experiments.
Data exclusions	No data exclusions.
Replication	*Describe the measures taken to verify the reproducibility of the experimental findings. If all attempts at replication were successful, confirm this OR if there are any findings that were not replicated or cannot be reproduced, note this and describe why.*
Randomization	Animals were randomly assigned for indicated treatments.
Blinding	Experimenters were not blinded to the assignments.

Reporting for specific materials, systems and methods

We require information from authors about some types of materials, experimental systems and methods used in many studies. Here, indicate whether each material, system or method listed is relevant to your study. If you are not sure if a list item applies to your research, read the appropriate section before selecting a response.

Materials & experimental systems

n/a	Involved in the study	
☐	☒	Antibodies
☒	☐	Eukaryotic cell lines
☒	☐	Palaeontology and archaeology
☐	☒	Animals and other organisms
☒	☐	Human research participants
☒	☐	Clinical data
☒	☐	Dual use research of concern

Methods

n/a	Involved in the study	
☒	☐	ChIP-seq
☐	☒	Flow cytometry
☒	☐	MRI-based neuroimaging

Antibodies

Antibodies used

Antibody used: CD16/32 FcBlock
Supplier name: eBioscience
Catalog number: 14-0161-82
Clone name: 93
Lot number: 207500

Antibody used: Fixable Viability Dye eFluor 506
Supplier name: eBioscience
Catalog number: 65086618
Lot number: 2198947

Antibody used: anti-Ly6G (GR-1) PE-Cy7
Supplier name: eBioscience
Catalog number: 13593185
Clone name: RB6-8C5
Lot number: E07648-1634

Antibody used: anti-Ly6G eFluor 450
Supplier name: eBioscience
Catalog number: 127612
Clone name: 1A8
Lot number: 13288476

Antibody used: anti CD117 FITC

Supplier name: eBioscience
Catalog number: 553354
Clone name: 2B8
Lot number: 86092

Antibody used: anti-SiglecF PE
Supplier name: eBioscience
Catalog number: 552126
Clone name: E50-2440
Lot number:

Antibody used: anti-CD11b PE-Dazzle 594
Supplier name: Biolegend
Catalog number: 101256
Clone name: M1/70
Lot number: B276558

Antibody used: anti-CD11c APC
Supplier name: Biolegend
Catalog number: 117310
Clone name: N418
Lot number: B262130

Antibody used: anti-MHC II (I-A/I-E) Alexa Fluor 700
Supplier name: eBioscience
Catalog number: 56532180
Clone name: M5/114.15.2
Lot number: 2118327

Antibody used: anti-CD49b PerCP eF710
Supplier name: eBioscience
Catalog number: 46597182
Clone name: DX5
Lot number: 2151530

Antibody used: anti-TCRβ APC-CY7
Supplier name: Biolegend
Catalog number: 109220
Clone name: H57-597
Lot number: B270114

Antibody used: anti-FceRIa PECy7
Supplier name: Biolegend
Catalog number: 134318
Clone name: MAR1
Lot number: B235750

Antibody used: anti-F4/80 APC/Cy7
Supplier name: Biolegend
Catalog number: 123118
Clone name: BM8
Lot number: B274179

Antibody used: anti-IL17A PerCP-Cy5.5
Supplier name: eBioscience
Catalog number: 45717782
Clone name: ebio 17B7
Lot number: 2082373

Antibody used: anti-CD4 Alexa Fluor 700
Supplier name: eBioscience
Catalog number: 56004182
Clone name: GK1.5

Antibody used: anti-CD4 eFluor450
Supplier name: eBioscience
Catalog number: 48004182
Clone name: GK1.5
Lot number: 1998362

Antibody used: anti-CD8α PE-CF594
Supplier name: BD Biosciences
Catalog number: 562283
Clone name: 53-6.7 (RUO)
Lot number:

Antibody used: anti-CD8α PerCP-Cy5.5
Supplier name: Tobno
Catalog number: 651886U100
Clone name: 2.43
Lot number: C1886020419653

Antibody used: anti-TCRδ APC
Supplier name: BioLegend
Catalog number: 118115
Clone name: GL3
Lot number: B2659570

Antibody used: anti-NK1.1 PECy7
Supplier name: BioLegend
Catalog number: 108714
Clone name: PK136
Lot number: B249074

Antibody used: anti-CD44 V500
Supplier name: BD Bioscience
Catalog number: 560780
Clone name: IM7
Lot number: 7324778

Antibody used: anti-CD62L PE-Cy7
Supplier name: BioLegend
Catalog number: 104418
Clone name: MEL-14
Lot number: B245457

Antibody used: anti-CD11b Alexa Fluor 647
Supplier name:
Catalog number:
Clone name:
Lot number:

Antibody used: anti-CD45R/B220 Alexa Fluor 700
Supplier name: BioLegend
Catalog number: 103232
Clone name: RA36B2
Lot number: B246157

Antibody used:PE-PBS57 loaded CD1d tetramer
Supplier name: National Institutes of Allergy and Infectious Diseases Tetramer Facility

Antibody used: anti-CD3
Supplier name: BD Biosciences

Antibody used: anti-CD28
Supplier name: BD Biosciences

Antibody used: inVivoMAb anti-Ly6G
Supplier name: BioXcell
Catalog number: BE0075-1
Clone name: 1A8
Lot number:

Antibody used: inVivoMAb anti-IgG2
Supplier name: BioXcell
Catalog number: BE0089-A025mg
Clone name: 2A3
Lot number:

Validation

anti-CD16/32 FcBlock: Jimenez RV, Kuznetsova V, Connelly AN, Hel Z, Szalai AJ. C-Reactive Protein Promotes the Expansion of Myeloid Derived Cells With Suppressor Functions. Front Immunol. 2019 Sep 18;10:2183. doi: 10.3389/fimmu.2019.02183. PMID: 31620123; PMCID: PMC6759522.

Fixable Viability Dye eFluor 506: manufacturer has certificate of analysis for lot number 2198947

anti-Ly6G (GR-1) PE-Cy7: manufacturer states "this Antibody was verified by Relative expression to ensure that the antibody binds to the antigen stated."

anti-Ly6G eFluor 450: Fleming TJ, et al. 1993. J. Immunol. 151:2399. (FC)

anti CD117 FITC: Anderson DM, Lyman SD, Baird A, et al. Molecular cloning of mast cell growth factor, a hematopoietin that is active

in both membrane bound and soluble forms. Cell. 1990; 63(1):235-243.

anti Siglec-F PE: Angata T, Hingorani R, Varki NM, Varki A. Cloning and characterization of a novel mouse Siglec, mSiglec-F: differential evolution of the mouse and human (CD33) Siglec-3-related gene clusters. J Biol Chem. 2001; 276(48):45128-45136.

anti-CD11b PE-Dazzle 594: Noel GJ, et al. 1990. J. Clin. Invest. 85:208

anti-CD11c APC: Granucci F, et al. 1997. J. Immunol. 159:1794.

anti-MHC II (I-A/I-E) Alexa Fluor 700 : Adam L, López-González M, Björk A, Pålsson S, Poux C, Wahren-Herlenius M, Fernández C, Spetz AL. Early Resistance of Non-virulent Mycobacterial Infection in C57BL/6 Mice Is Associated With Rapid Up-Regulation of Antimicrobial Cathelicidin Camp. Front Immunol. 2018 Sep 3;9:1939. doi: 10.3389/fimmu.2018.01939. PMID: 30233570; PMCID: PMC6129578.

anti-CD49b PerCP eF710: Krneta T, Gillgrass A, Chew M, Ashkar AA. The breast tumor microenvironment alters the phenotype and function of natural killer cells. Cell Mol Immunol. 2016 Sep;13(5):628-39. doi: 10.1038/cmi.2015.42. Epub 2015 Aug 17. PMID: 26277898; PMCID: PMC5037278.

anti-TCRβ APC-CY7 : Tsukumo S, et al. 2006. J.Immunol. 177:8365.

anti-FceRIa PECy7: Obata K, et al. 2007. Blood 110:913

anti-F4/80 APC/Cy7: Herbst S, et al. 2013. Dis Model Mech. 6:643.

anti-IL17A PerCP-Cy5.5: Lin W, Wang N, Zhou K, Su F, Jiang Y, Shou J, Liu H, Ma C, Qian Y, Wang K, Wang X. RKIP mediates autoimmune inflammation by positively regulating IL-17R signaling. EMBO Rep. 2018 Jun;19(6):e44951. doi: 10.15252/embr.201744951. Epub 2018 Apr 19. Erratum in: EMBO Rep. 2019 Feb;20(2): PMID: 29674348; PMCID: PMC5989851.

anti-CD4 Alexa Fluor 700: manufacturer website states "This Antibody was verified by Relative expression to ensure that the antibody binds to the antigen stated."

anti-CD4 eFluor450: manufacturer website states "This Antibody was verified by Relative expression to ensure that the antibody binds to the antigen stated."

anti-CD8α PE-CF594: Bierer BE, Sleckman BP, Ratnofsky SE, Burakoff SJ. The biologic roles of CD2, CD4, and CD8 in T-cell activation. Annu Rev Immunol. 1989; 7:579-599.

anti-CD8α PerCP-Cy5.5: Willinger T and Flavell RA. 2012. Proc. Natl. Acad. Sci. 109:8670-8675.

anti-TCRδ APC: Kasten KR, et al. 2010. Infect. Immun. 78:4714

anti-NK1.1 PECy7: Carlyle JR, Martin A, Mehra A, Attisano L, Tsui FW, Zuniga-Pflucker JC. Mouse NKR-P1B, a novel NK1.1 antigen with inhibitory function. J Immunol. 1999; 162(10):5917-5923.

anti-CD44 V500: Bendelac A. Mouse NK1+ T cells. Curr Opin Immunol. 1995; 7(3):367-374.

anti-CD62L PE-Cy7: Shigeta A, et al. 2008. Blood 112:4915

anti-CD11b Alexa Fluor 647:

anti-CD45R/B220 Alexa Fluor 700: Shih FF, et al. 2006. J. Immunol. 176:3438.

PE-PBS57 loaded CD1d tetramer: NIH Tetramer Core Facility website states: "PBS-57 is an analogue of a-galactosylceramide developed by Dr. Paul Savage and colleagues. Three independent laboratories have shown that PBS-57 activity is indistinguishable from a-galactosylceramide. The NIH Tetramer Facility provides PBS-57 ligand complexed to CD1d monomers or tetramers. This is the recommended reagent for detection of NKT cells."

anti-CD3:

anti-CD28:

inVivoMAb anti-Ly6G: Davis, R. W. t., et al. (2018). “Luminol Chemiluminescence Reports Photodynamic Therapy-Generated Neutrophil Activity In Vivo and Serves as a Biomarker of Therapeutic Efficacy.”

inVivoMAb anti-IgG2: Bauche, D., et al. (2018). “LAG3(+) Regulatory T Cells Restrain Interleukin-23-Producing CX3CR1(+) Gut-Resident Macrophages during Group 3 Innate Lymphoid Cell-Driven Colitis.” Immunity 49(2): 342-352 e345.

Animals and other organisms

Policy information about studies involving animals; ARRIVE guidelines recommended for reporting animal research

Laboratory animals	Species: Mus musculus<br><br>Strain: All mice were on a C57Bl/6 background. We used the following mice: . Itk-/-, B6.129P2-Tcrdtm1Mom/J, C57BL/6-Il17atm1Bcgen/J and C57BL/6-Foxp3tm1Flv/J to generate IL17A-GFP/Foxp3-RFP reporter strains in WT or Itk-/- background.<br><br>Sex: Female and Male<br><br>Age: 6-8 weeks
Wild animals	The study did not involve wild animals.
Field-collected samples	This study did not involve samples collected from the field.
Ethics oversight	All experiments were approved by the Office of Research Protection’s Institutional Animal Care and Use Committee at The Pennsylvania State University and Cornell University.

Note that full information on the approval of the study protocol must also be provided in the manuscript.

Flow Cytometry

Plots

Confirm that:

☒ The axis labels state the marker and fluorochrome used (e.g. CD4-FITC).

☒ The axis scales are clearly visible. Include numbers along axes only for bottom left plot of group (a 'group' is an analysis of identical markers).

☒ All plots are contour plots with outliers or pseudocolor plots.

☒ A numerical value for number of cells or percentage (with statistics) is provided.

Methodology

Sample preparation	Cells were collected from the following organs: spleen, lung, draining lymph node, and bronchial lavage. Cells were isolated from spleen and draining lymph node were minced and passed through 70 micron cell strainer in RPMI. Lung samples were minced and digested in RMPIfor 15 to 30 minutes at 37°C then passed through 70 micron cell strainer. Red blood cells were lysed with ammonium chloride potassium buffer.
Instrument	BD LSR II Special Order System<br>BD Biosciences FACSymphony<br>BDFACSAria Fusion Cell Sorter
Software	Software used to collect flow cytometry data was BD Diva and FlowJo and GraphPad Prism was used to analyze flow cytometry data.
Cell population abundance	Cell type: WT CD44+TCRb+CD4+Foxp3+IL10+<br>Abundance (cells counts): 39,500<br>Purity (determined by flow cytometry):

Cell type:
Abundance (cells counts):
Purity (determined by flow cytometry):

Cell type:
Abundance (cells counts):
Purity (determined by flow cytometry):

Cell type:
Abundance (cells counts):
Purity (determined by flow cytometry):

Cell type:
Abundance (cells counts):
Purity (determined by flow cytometry):

Gating strategy

Cells were gated on lymphocyte or granulocyte populations based on their respective experiments via FSC and SSC. Doublet and dead cells were gated out by using Viability dye .

To determine the population of γδ or αβ T cells, cells were gated on TcRαβ vs TCRγδ followed by gating for αβ T cell subsets CD4 and CD8.

To determine the population of neutrophils, cells were gated on CD11b v Ly6G?

To determine IL17A-GFP+ expression, cells were gated for FSC vs GFP, then then TcRαβ vs TCRγδ followed by gating for αβ T cell subsets CD4 and CD8 then analyzed for GFP expression.

To determine the neutrophil population in the neutrophil depletion experiment, neutrophils were gated on Ly6G vs GR1.

☒ Tick this box to confirm that a figure exemplifying the gating strategy is provided in the Supplementary Information.
